# BRD4 inhibitor JQ1 inhibits and reverses mechanical injury-induced corneal scarring

**DOI:** 10.1038/s41420-018-0066-1

**Published:** 2018-06-28

**Authors:** Mingli Qu, Xiaoping Zhang, Xiaoli Hu, Muchen Dong, Xiaojing Pan, Jiang Bian, Qingjun Zhou

**Affiliations:** 1 State Key Laboratory Cultivation Base, Shandong Provincial Key Laboratory of Ophthalmology, Shandong Eye Institute, Shandong Academy of Medical Sciences, Qingdao, China; 2 The Affiliated Hospital of Qingdao University, Qingdao, Shandong China; 3 School of Medicine and Life Sciences, University of Jinan-Shandong Academy of Medical Sciences, Jinan, Shandong China

**Keywords:** Mechanisms of disease, Gene silencing

## Abstract

Corneal scarring is characterized by the improper deposition of extracellular matrix components and myofibroblast differentiation from keratocytes. The bromodomain-containing protein 4 (BRD4) inhibitor JQ1 has been shown to attenuate pathological fibrosis. The present study aimed to explore the potential therapeutic effect of JQ1 on mechanical injury-induced mouse corneal scarring and TGFβ-induced human corneal myofibroblast differentiation and the related mechanism. The corneal scarring and myofibroblast differentiation were evaluated with clinical observation and fibrosis-related gene expression analysis. In mice, subconjunctivally injected JQ1 suppressed the initial development and reversed the established progression of corneal scarring, while having no impairment on the epithelial regenerative capacity. BRD4 inhibition with either JQ1 or small-interfering RNA inhibited the differentiation and promoted the dedifferentiation of human corneal myofibroblasts. Moreover, JQ1 attenuated the accumulation of intracellular reactive oxygen species induced by TGFβ treatment, induced Nrf2 nuclear translocation and activated the expression of Nrf2-ARE downstream antioxidant genes. In conclusion, this study implicates that JQ1 suppresses and reverses corneal scarring through the regulation of BRD4 inhibition and Nrf2-dependant antioxidant induction.

## Introduction

Corneal transparency is important for the light transmission to the retina for optimal vision. In human eyes, corneal scarring is the most common result after trauma, infection, or refractive surgery and causes visual impairment^1^. Keratocytes, the major cellular elements in the corneal stroma, remain quiescent and synthesize extracellular matrix (ECM) components to maintain corneal stromal turnover. Following injury, inflammation-induced transforming growth factor β (TGFβ) activates the transition of corneal keratocytes into fibroblasts and myofibroblasts with the expression of α-smooth muscle actin (α-SMA) as key marker^2–5^. Myofibroblasts rapidly synthesize and secrete redundant ECM proteins, including Collagen I and fibronetin during wound healing, leading to the development of corneal scarring and opacity^6^. Currently, targeting therapies primarily depend on corneal transplantation. Pharmacologic interventions with corticosteroids and mitomycin C are limited because of the side effects such as ulceration and reduced keratocyte density of the anterior stroma^7–9^.

Bromodomain-containing protein 4 (BRD4), a bromodomain and extraterminal family member and an important epigenetic reader by binding to acetylated lysines, was reported to colocalize with profibrotic transcription factors and was identified as a probable driver of fibrosis reaction^10–12^. From the therapeutic viewpoint, molecules that can block the fibrogenic effects of BRD4 are of great clinical interests. The prototype compound JQ1 is a small-molecule inhibitor of BRD4 that disturbs the binding of BRD4 to acetylated lysines. Recently, JQ1 was verified to attenuate various tissue fibroses, including hepatic, renal and heart fibroses, both in vivo and in vitro^12–14^. However, the role of JQ1 in corneal scarring remains unknown.

Herein, we explored the potential therapeutic effect of JQ1 on corneal scarring and corneal myofibroblast differentiation, finding that JQ1 dramatically inhibited and reversed TGFβ-induced fibrotic gene expression and F-actin bundle formation in cultured human corneal fibroblasts (HCFs). Moreover, JQ1 suppressed the initial development and reversed the established corneal scarring in mice. The inhibitory effect of JQ1 on corneal scarring was related to the BRD4 inhibition and antioxidant activity.

## Results

### JQ1 inhibits TGFβ-induced corneal myofibroblast differentiation

**Fig. 1:**
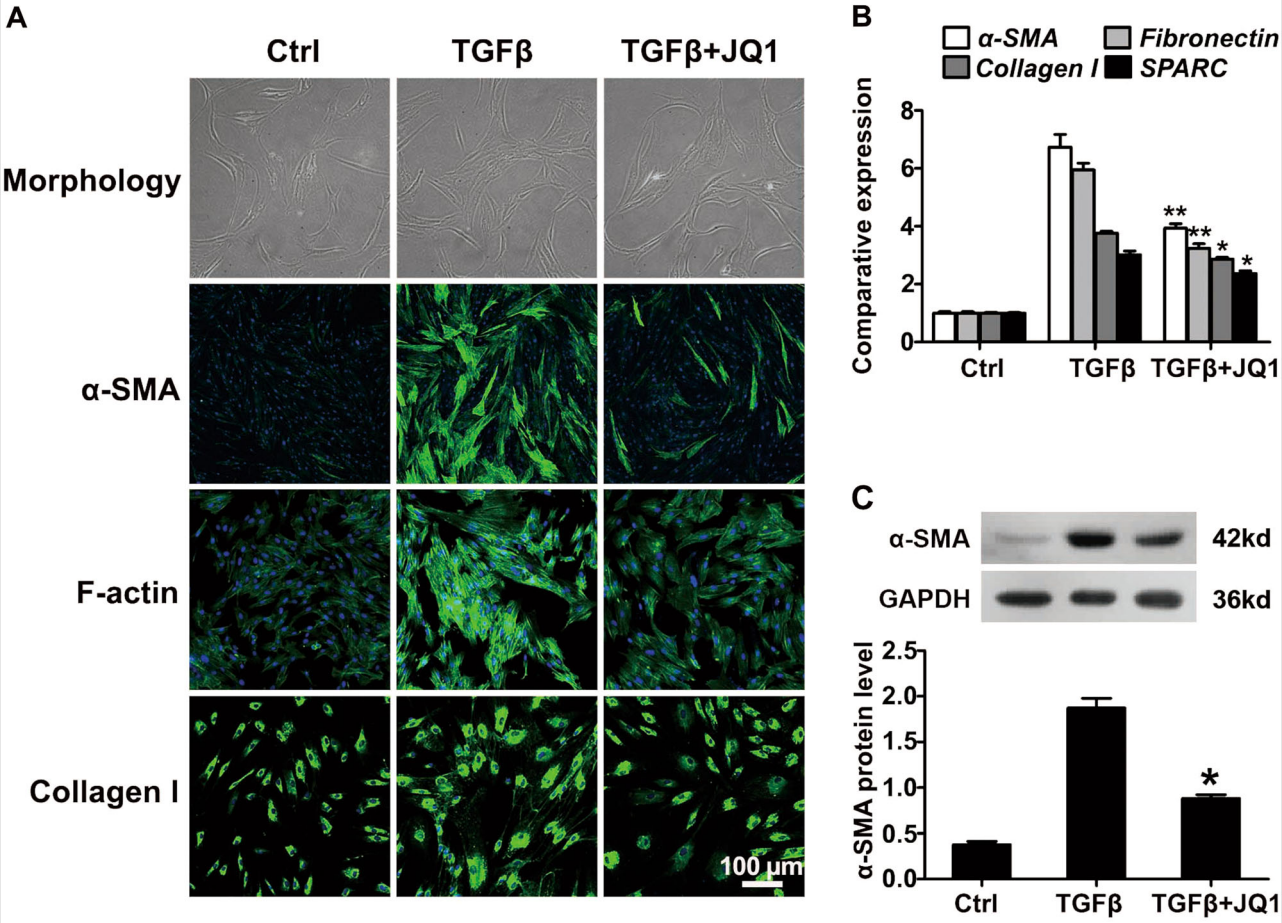
JQ1 inhibits TGFβ-induced corneal myofibroblast differentiation. Human corneal fibroblasts were treated with TGFβ (2 ng/ml) and JQ1 (200 nM) for 3 days. **a** Representative images of cellular morphology, immunofluorescence staining of α-SMA, F-actin and Collagen I. **b** Real-time PCR analyses for the mRNA expression of the indicated genes. **c** Western blot analysis for the protein expression of α-SMA. *P < 0.05, **P < 0.01 vs. the TGFβ group. The data are shown as means ± SEM

Primary HCFs were treated with 2 ng/ml TGFβ in the presence or absence of 200 nM JQ1 to evaluate the inhibitory effect of JQ1 on myofibroblast differentiation. Immunofluorescence staining results showed that TGFβ treatment caused the corneal myofibroblast differentiation with enlarged morphology, enhanced staining of α-SMA, F-actin and Collagen I. However, the JQ1 supplement suppressed the differentiation of corneal myofibroblasts induced by TGFβ, which is shown as the weaker staining of α-SMA, F-actin and Collagen I (Fig. 1a). Real-time PCR and western blot results further confirmed the inhibitory effect of JQ1 on corneal myofibroblast differentiation, including the decreased mRNA levels of *α-SMA*, *Fibronectin*, *Collagen I* and *SPARC*, and the protein level of α-SMA, when compared to the cells with TGFβ treatment (Fig. 1b, c).

### JQ1 attenuates mechanical injury-induced corneal scarring

**Fig. 2:**
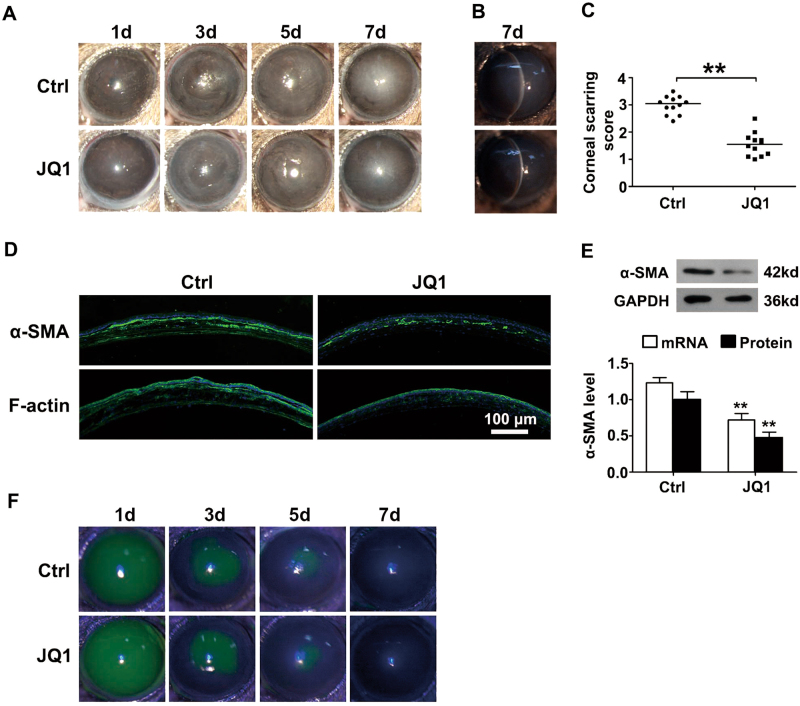
JQ1 attenuates mechanical injury-induced corneal scarring. JQ1 (1 mM in 7 µl) or PBS as vehicle control was injected subconjunctivally 1 h post injury. **a** Representative bright-field microscopic images of the injured cornea. **b** Slit-lamp photograph of the corneal stromal opacity of the control and JQ1-treated mice at 7 days post injury. **c** The corneal scarring score was graded on a scale of 0–4 (*n* = 12) at 7 days. **d** Representative images of immunofluorescence staining of α-SMA and F-actin at 7 days. **e** Total RNA was isolated from the harvested corneas at 7 days post injury, real-time PCR and Western blot were performed to analyze the expression of α-SMA. **f** The size of the corneal epithelial defect in JQ1-treated mice and the control group. **P < 0.01, relative to the control group. The data are shown as means ± SEM

To investigate the effect of JQ1 during the progression of corneal scarring, a mechanical injury-induced mouse corneal scarring model was established by removing the corneal epithelium and anterior stroma. Photographs of injured corneas were captured using slit-lamp biomicroscopy with or without fluorescein staining at 1, 3, 5 and 7 days post injury. The central and disk-shaped scarring was remarkable at 7 days in the control group (Fig. 2a). However, JQ1-treated mice exerted alleviated corneal scarring with a significantly lower scarring score when compared to the control group at 7 days post injury (*n* = 12; Fig. 2b, c), immunofluorescence staining showed the reduced staining of α-SMA and F-actin in JQ1-treated corneas compared to the control group (Fig. 2d), which was further confirmed by the decreased mRNA and protein expression of α-SMA (Fig. 2e). Moreover, fluorescein staining showed the corneal epithelial healing was not impaired with the subconjunctivally injected JQ1 (Fig. 2f). The results suggest that JQ1 treatment attenuates mechanical injury-induced corneal scarring in mice, with no toxic effect on epithelial healing.

### JQ1 induces the dedifferentiation of corneal myofibroblasts

**Fig. 3:**
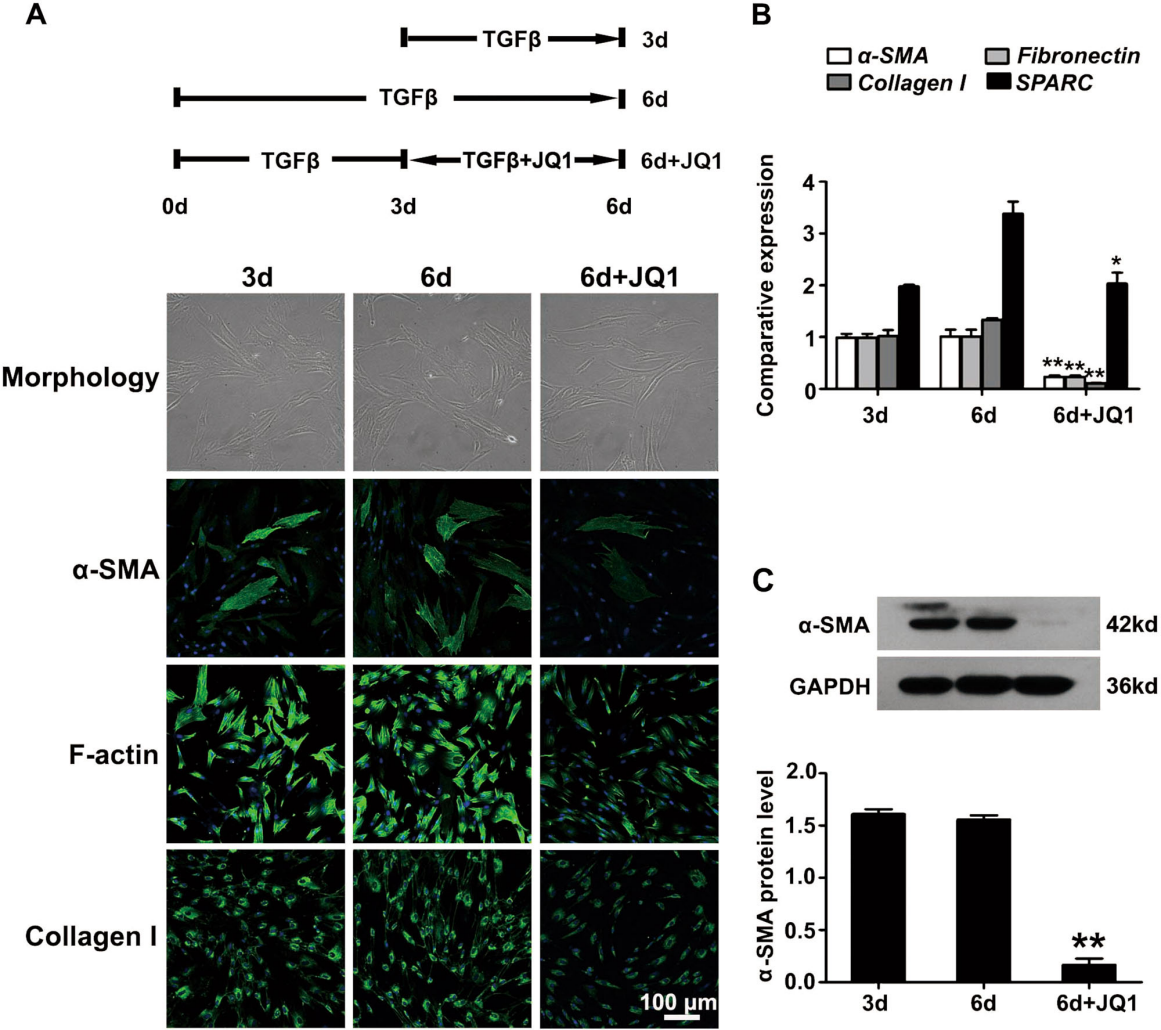
JQ1 induces the dedifferentiation of corneal myofibroblasts. Human primary corneal fibroblasts were treated with 2 ng/ml TGFβ for 3 and 6 days, and with 2 ng/ml TGFβ for 6 days plus 200 nM JQ1 from day 4, respectively. **a** Representative images of cellular morphology, immunofluorescence staining of α-SMA, F-actin and Collagen I after the addition of JQ1 for 3 days when compared to the cells treated with TGFβ only. **b** Real-time PCR analyses for the mRNA expression of the indicated genes. **c** Western blot analysis for the protein expression of α-SMA. *P < 0.05, **P < 0.01 vs. the TGFβ-6d group. The data are shown as means ± SEM

To verify whether JQ1 could induce the dedifferentiation of corneal myofibroblasts in vitro, primary HCFs were treated with 2 ng/ml TGFβ for 3 and 6 days, and with TGFβ for 6 days plus JQ1 for the later 3 days, respectively (Fig. 3a). Immunofluorescence staining, real-time PCR and western blot demonstrated the complete differentiation of corneal myofibroblasts treated by TGFβ for 3 days, which assumed the identical changes with those treated by TGFβ for 6 days (Fig. 3a–c). However, the differentiated corneal myofibroblasts reversed to the fibroblastic morphology, with weaker expressions of α-SMA, F-actin, Collagen I and *SPARC*, and a decreased protein level of α-SMA after the later treatment with JQ1 for 3 days when compared to the cells solely treated with TGFβ for 3 or 6 days (Fig. 3a–c). Taken together, these results suggest that JQ1 treatment can not only inhibit myofibroblast differentiation but also induce the dedifferentiation of corneal myofibroblasts in vitro.

### JQ1 reverses the established corneal scarring in mice

**Fig. 4:**
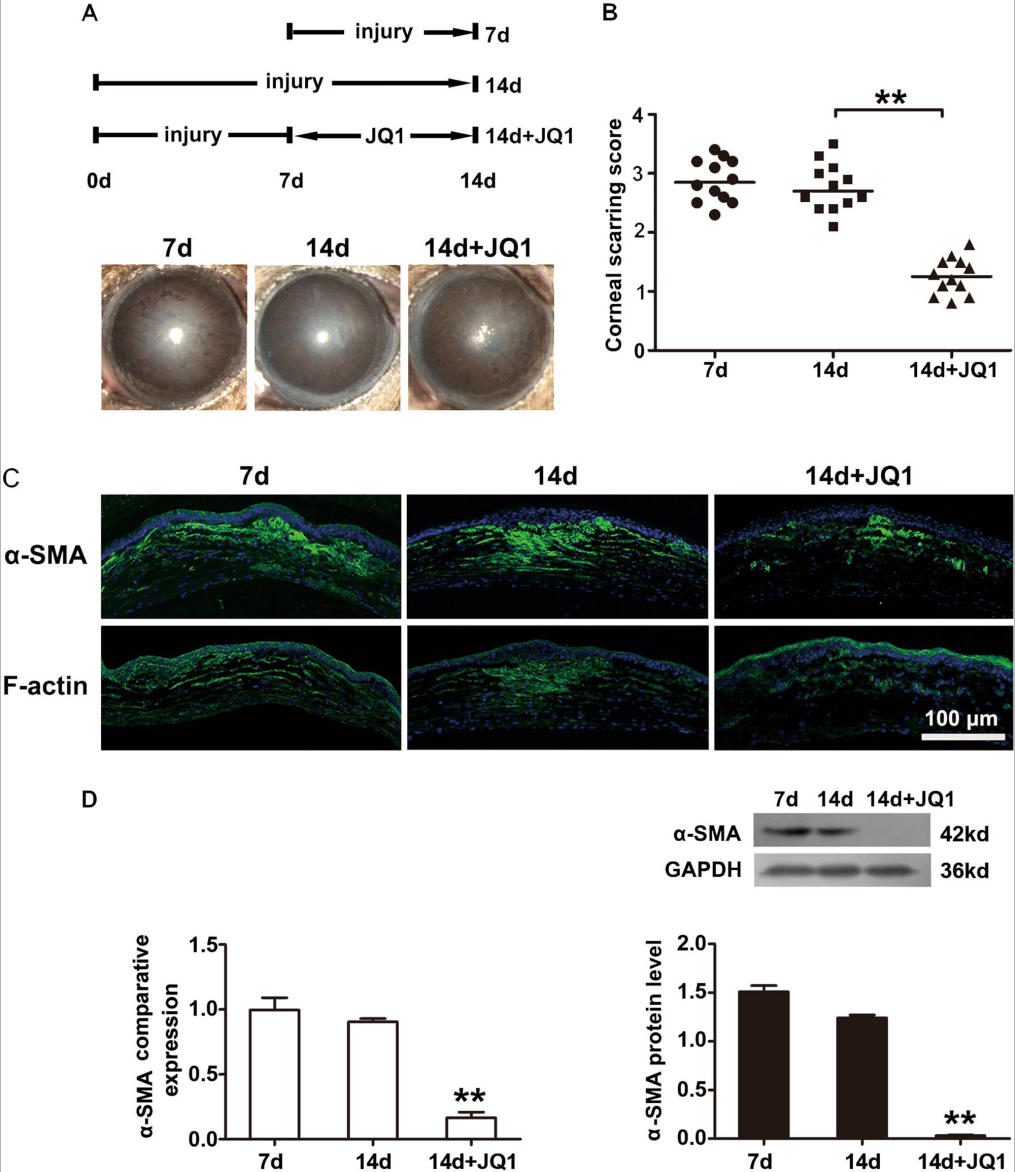
JQ1 reverses the progression of established corneal scarring in mice. JQ1 (1 mM in 7 µl) or PBS as vehicle control was injected subconjunctivally from day 7 after mechanical injury, when the corneal scarring had been established. **a** Representative bright-field microscopic images of the cornea scarring model. **b** The corneal scarring score was graded on a scale of 0 to 4 (*n* = 12). **c** Typical images of immunofluorescence staining of α-SMA and F-actin. **d** Real-time PCR and western blot analyses of α-SMA for the harvested corneas. **P < 0.01, relative to the 14-day group. The data are shown as means ± SEM

To further investigate whether JQ1 can reverse the established corneal scarring in vivo, the mice with corneal scarring were treated with JQ1 for 7 days from day 7 after mechanical injury, when the corneal scarring had been established (Fig. 4a). The mice with 7-day JQ1 treatment presented an alleviated corneal scarring with a significantly lower scarring score when compared to the status at 7 and 14 days post injury (*n* = 12; Fig. 4b). Immunofluorescence staining revealed weaker staining of α-SMA and F-actin in the JQ1-treated corneas than the untreated groups, which was further confirmed by the decreased mRNA and protein expression of α-SMA (Fig. 4c, d). Taken together, these results suggest that JQ1 reverses the established corneal scarring in vivo.

### JQ1 blocks corneal myofibroblast differentiation via BRD4 inhibition

**Fig. 5:**
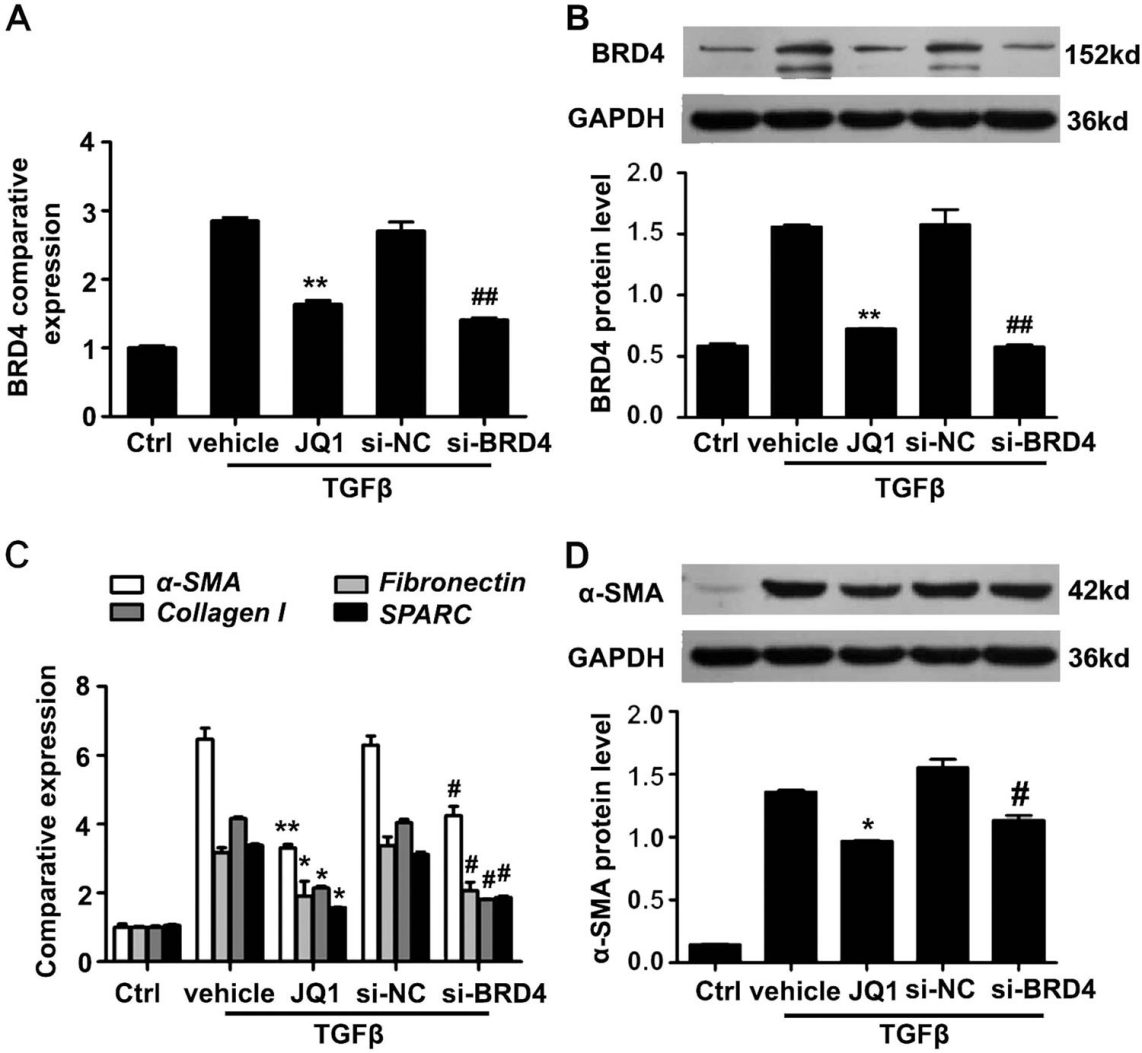
JQ1 blocks corneal myofibroblast differentiation via BRD4 inhibition. Telomerase-immortalized human corneal fibroblasts (HTKs) were transfected with 1 µg BRD4 siRNA or control siRNA, and treated with TGFβ (5 ng/ml) with or without JQ1 (200 nM) for 24 h. **a** Real-time PCR analysis and **b** western blot detection of the BRD4 knockdown efficiency. **c** The expression of the indicated genes after BRD4 knockdown or JQ1 administration in TGFβ treated HTKs. **d** Western blot analysis for the protein expression of α-SMA in the indicated groups. *P < 0.05, **P < 0.01 vs. TGFβ group; ^#^*P* < 0.05^##^,*P* < 0.01 vs. si-NC group. Data are shown as means ± SEM

As JQ1 is a small-molecule inhibitor of BRD4, we hypothesized that BRD4 played an important role in modulating corneal myofibroblast differentiation. In support of this view, JQ1 administration and siRNA transfection methods were used to regulate the expression of BRD4 in telomerase-immortalized human corneal fibroblasts (HTK cell line, presented by Prof. Jester of University of California, Irvine). Following siRNA-transfection, the mRNA and protein expression of BRD4 was significantly declined and reached the same levels to JQ1 treatment (Fig. 5a, b). Similar to JQ1 administration, siRNA-mediated inhibition of BRD4 markedly reduced the mRNA level of *α-SMA*, *Fibronectin*, *Collagen I* and *SPARC*, and the protein level of α-SMA in TGFβ-treated HTKs (Fig. 5c, d). These results imply that the inhibition of BRD4 is an effective therapy to suppress myofibroblast differentiation.

### JQ1 attenuates intracellular reactive oxygen species accumulation and activates Nrf2-ARE signaling

**Fig. 6:**
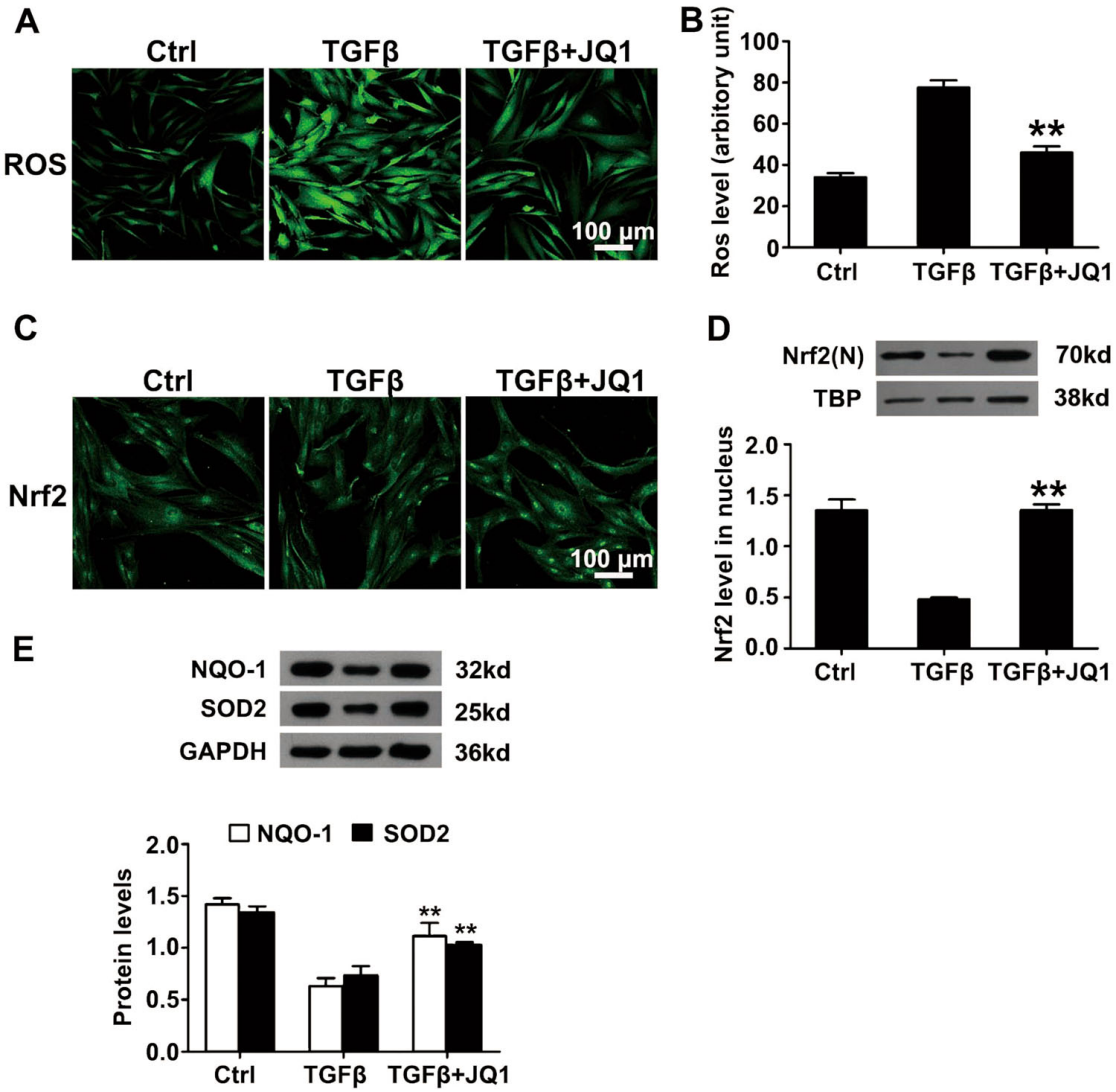
JQ1 attenuates intracellular ROS accumulation and activates Nrf2-ARE signaling. Human corneal fibroblasts were treated with TGFβ (2 ng/ml) and JQ1 (200 nM) for 3 days. **a** ROS staining in corneal fibroblasts treated with TGFβ and JQ1. **b** Absorbance measurement of ROS in the indicated groups. **c** Nuclear accumulation of Nrf2 based on immunofluorescence staining. **d** The Nrf2 level in the nucleus based on the Western blot analysis. **e** The expression of the Nrf2-ARE downstream antioxidant protein including NQO1 and SOD2 in the indicated groups. Nrf2 (N) indicates Nrf2 in the nucleus. *P < 0.05, **P < 0.01 vs. TGFβ group. Data are shown as means ± SEM

Previous studies have confirmed that reactive oxygen species (ROS) and Nrf2-ARE signaling played a role in corneal myofibroblast differentiation^15,16^. On this basis, we examined whether JQ1 can regulate the ROS accumulation induced by TGFβ treatment in corneal myofibroblast differentiation. Representative results showed weaker staining and reduced accumulation of ROS with JQ1 treatment than vehicle control in the presence of TGFβ treatment (Fig. 6a, b). Moreover, JQ1 treatment enhanced the staining of Nrf2 in the nucleus and promoted nuclear Nrf2 translocation, as confirmed by western blot (Fig. 6c, d). In addition, 3 days of JQ1 treatment efficiently reactivated the expression of TGFβ-suppressed antioxidant genes, including NQO1 and SOD2 (Fig. 6e). These results suggest that JQ1 promotes the accumulation of Nrf2 in the nucleus from the cytoplasm and possibly results in activating the transcription of antioxidant genes.

## Discussion

As a novel therapeutic target of fibrosis, BRD4 was reported to implicate in fibrosis process by regulating fibrosis gene expression, indicating that BRD4 inhibition played an important role in the suppression of myofibroblast differentiation and organ fibrosis^17–19^. In our study, we demonstrated that JQ1 suppressed the TGFβ-induced α-SMA, Fibronectin, Collagen I and SPARC expressions in HCFs and prevented their differentiation to myofibroblasts, which is the initial reason of corneal scarring^4,20^. Then, our in vivo studies showed that subconjunctival injection of JQ1, the BRD4 inhibitor, restrained corneal scarring induced by mechanical injury in mice. We also further indicated that JQ1 could reverse the established progression of corneal scarring in vivo and induce the dedifferentiation of myofibroblasts in vitro. Moreover, the siRNA knockdown results confirmed the role of BRD4 in suppressing TGFβ-induced fibrosis gene expression in HTKs. JQ1 application was also found to alleviate TGFβ-induced intracellular oxidative stress and promote the activation of Nrf2-ARE signaling in HCFs. All these results bring up the novel evidence that JQ1 suppresses and reverses corneal scarring through the regulation of BRD4 inhibition and Nrf2-dependant antioxidant induction.

Fibrosis is related to many disorders, such as chronic kidney disease, liver cirrhosis, heart failure, idiopathic pulmonary fibrosis, radiation-induced fibrosis and corneal scarring^6,11,21–25^. Besides the conventional therapies like keratoplasty and immunosuppressive steroids intervention, new therapeutic methods have been used to treat ocular damage caused by corneal scarring, including the inhibition of mammalian target of rapamycin (mTOR), Tichostatin a, galectin-3, and the suppression of MSCs or HGF inducted by IL-1β^9,26–28^. Recently, BRD4 was reported to be a novel therapeutic target for various fibrosis diseases by suppressing the expression of related genes^29^. JQ1, a main inhibitor of BRD4, was demonstrated to play a part in anti-fibrosis in many tissues and organs, like liver, kidney, lung and heart^10,12–14^, in addition to its well accepted effects in suppressing cancer proliferation, inflammation and bone destruction^30–32^. Our work disclosed that subconjunctivally injected JQ1 could not only suppress the initial development but also reverse the established progression of corneal scarring and myofibroblast differentiation.

Previous studies had confirmed the role of accumulated intracellular ROS induced by TGFβ in various fibrosis-related diseases^33–35^. TGFβ together with its downstream NADPH oxidase 4 amplifies the oxidative stress signaling and thus enhances the fibrogenic program^36–38^. As an antioxidation-related transcription factor, cytoplasmic Nrf2 is highly unstable when it bounds to Kelch-like ECH-associated protein 1 (Keap1) and thus results in Nrf2 selective proteasomal degradation^39,40^. However, under oxidative stress condition, Nrf2 escapes from the Nrf2-Keap1 complex and transports into the nucleus^41,42^, leading to an increased expression of Nrf2-ARE downstream antioxidant genes, including NADPH quinine oxidoreductase-1 (NQO1) and superoxide dismutase(SOD)^43^. Recent evidences have revealed that BRD4 promotes the transcription of Nrf2 inhibition protein KEAP1, so that the inhibition of BRD4 by JQ1 reactivates the transcription of Nrf2-dependent genes and alleviates oxidative stress in many in vitro cell lines, like human airway smooth muscle cells, human monocytic cells, and human embryonic kidney 293 cells^44,45^. Our in vitro study in HCFs further indicated that JQ1 application attenuated TGFβ-induced intracellular ROS accumulation by promoting the activation of Nrf2-ARE signaling, which finally led to the inhibition of corneal myofibroblast differentiation. Our findings bring forward a novel working model of JQ1 on anti-fibrosis partly through the activation of Nrf2-dependent anti-ROS pathway in HCFs, which might serve as the potential mechanism of JQ1 treatment of corneal scarring.

In conclusion, we identified that JQ1 administration not only inhibited the myofibroblastic differentiation and corneal scarring formation, but also induced myofibroblastic dedifferentiation and established corneal scarring attenuation. The downstream Nrf2-ARE signaling was implicated in mediating the effect of JQ1 on suppressing and reversing mechanical injury-induced corneal scarring. Therefore, our study indicates that BRD4 is a potential therapeutic target for corneal fibrosis, and JQ1 intervention serves as an attractive therapy for corneal haze or scar resolution.

## Materials and methods

### Animals

Male C57BL/6 mice (6–8 weeks) were purchased from the Beijing Pharmacology Institute (Beijing, China) and maintained in the animal center of Shandong Eye Institute. All animal experiments were performed in accordance with the ARVO Statement for the Use of Animals in Ophthalmic and Vision Research and approved by the Institutional Review Board of Shandong Eye Institute. The corneal scarring model was prepared according to previous reports with minor modifications^28,46^. In brief, under systemic and topical anesthesia, the mouse corneal epithelium (3 mm in diameter) and anterior stromal portion were removed with the algerbrush II corneal rust ring remover (Alger Co, Lago Vista, TX, USA) and a razor blade, respectively. JQ1 (1 mM in 7 µl; Abcam, Cambridge, MA, USA) or PBS as vehicle control was injected subconjunctivally 1 h post injury. Finally, the mice were topically administered with ofoxacin eye drops (Santen, Osaka, Japan) to avoid infection. Only one eye was used in each animal for all experiments. All eyes were photographed under slit lamp (BQ900; Haag-Streit, Bern, Switzerland) at 1, 3, 5 and 7 days after the mechanical injury for the grading of corneal scarring (*n* = 12). The corneal scarring was graded on a scale of 0–4 (0, completely clear; 0.5, minimal scarring with a careful oblique illumination; 1, mild scarring not interfering with visibility of fine iris details; 2, mild opacification of iris details; 3, moderate opacification of the iris and lens; 4, complete opacification of the anterior chamber and iris)^26^. To evaluate the potential toxic effect of JQ1 on corneal epithelial regeneration, 0.25% fluorescein sodium was used to stain the defects of corneal epithelium, and photographs were taken at 1, 3, 5, 7 days after injury. Mouse corneas were collected for real-time PCR, immunofluorescence staining and Western blot analysis.

### Cell culture

Human corneal fibroblasts (HCFs) were isolated and cultured as previously reported^4^. To evaluate the inhibitory effect of JQ1 in myofibroblast differentiation, HCFs were treated with 2 ng/ml TGFβ (R&D Systems, Minneapolis, MN, USA) for 3 days with or without 200 nM JQ1. To evaluate the reversal effect of JQ1 on corneal myofibroblasts, HCFs were treated with 2 ng/ml TGFβ for 3 days followed by 200 nM JQ1 treatment for additional 3 days. All cells were further analyzed for α-SMA, Fibronectin, Collagen I, and SPCRC expressions, F-actin bundle formation, ROS accumulation, Nrf2 and Nrf2-ARE downstream antioxidant gene expressions. The concentrations of TGFβ and JQ1 were optimized according to our preliminary experiment.

### Transfection of siRNAs

Telomerase-immortalized human corneal fibroblasts (HTK cell line) were cultured as previously described^43^. HTKs (2 × 10^5^ cells) were seeded and transfected with 1 µg BRD4 siRNA (sc-43639; Santa Cruz Biotechnology, Santa Cruz, CA, USA) or control siRNA duplex (sc-37007; Santa Cruz Biotechnology). After 5–7 h incubation, the transfection medium was replaced with normal medium for 40 h, the cells were treated with 5 ng/ml TGFβ with or without 200 nM JQ1 for 24 h. Then, the cells were collected for the extraction of total RNA and total proteins. The expressions of BRD4 and fibrotic genes were detected at both the mRNA and protein levels.

### Real-time qPCR

Total RNA was extracted from corneal fibroblasts or corneal tissues using the NucleoSpin RNA kit (BD Biosciences, Palo Alto, CA, USA). Isolated RNA was reversely transcribed into cDNA using the PrimeScript first-strand cDNA synthesis kit (TaKaRa, Dalian, China). Real-time PCR was carried out using SYBR Green reagents and the Applied Biosystems 7500 Real-Time PCR System (Applied Biosystems, Foster City, CA, USA). The specific primers used in this assay are listed in Table 1. The results were analyzed with the Sequence Detection System (SDS) software (Applied Biosystems) using GAPDH as an internal control.

**Table 1:** Nucleotide sequences of primers used for RT-qPCR

Genes	Forward primer (5′–3′)	Reverse primer (5′–3′)	Gene accession
h-*α-SMA*	GGTGACGAAGCACAGAGCAA	CAGGGTGGGATGCTCTTCAG	NM_001141945.2
h-*fibronectin*	GGGACCGTCAGGGAGAAAA	CGAGATATTCCTTCTGCCACTGTT	NM_212482.2
h-*collagen I*	TTGTGCGATGACGTGATCTGT	TTGGTCGGTGGGTGACTCTG	NM_000088.3
h-*SPARC*	GGCTTCTCCTCCTCTGTCTT	AACCGATTCACCAACTCCAC	NM_003118.3
h-*BRD4*	CTAAACTGGAGGCCCGTGAGT	CAAAGCGCATTTCGAACACA	NM_014299.2
h-*GAPDH*	ATGCTGGCGCTGAGTACGT	AGCCCCAGCCTTCTCCAT	NM_002046.4
m-*α-SMA*	TGCCGAGCGTGAGATTGTC	CGTTCGTTTCCAATGGTGATC	NM_007392.3
m-*GAPDH*	GATGCCCCCATGTTTGTGAT	GGCATGGACTGTGGTCATGAG	NM_008084.2

### Immunofluorescence staining

Cultured cells were fixed with 4% paraformaldehyde for 10 min at room temperature. Mouse corneal cryosections were fixed with ice methanol for 10 min at −20 °C. All samples were blocked with 5% BSA (Sigma-Aldrich Corp, St. Louis, MO, USA) and incubated with FITC-conjugated phalloidin (Alexis Biochemicals, San Diego, CA, USA), rabbit anti-α-SMA (Abcam), rabbit anti-Collagen I (Abcam) and rabbit anti-Nrf2 (Abcam) antibodies at 4 °C overnight. The samples were subsequently incubated with Alexa Fluor 488-labeled donkey anti-rabbit IgG antibody (Invitrogen, Carlsbad, CA, USA) at 37 °C for 1 h. Nuclear counterstaining was performed using 4′,6-diamidino-2-phenylindole (DAPI, Sigma-Aldrich). Finally, the staining was observed under a confocal laser-scanning microscope (Nikon, Tokyo, Japan) or a fluorescence microscope (Nikon).

### Western blot analysis

Cultured cells or mouse corneal tissues were lysed with RIPA buffer containing protease inhibitors (Beyotime, Jiangsu, China). The protein samples were separated on SDS polyacrylamide gels and transferred to PVDF membranes (Millipore, Billerica, MA, USA). The blots were blocked by non-fat dry milk for at least 1 h, and incubated with rabbit anti-α-SMA (Abcam), rabbit anti-BRD4 (Abcam), rabbit anti-Nrf2 (Abcam), mouse anti-TBP (Abcam), rabbit anti-SOD2 (Abcam) and rabbit anti-NQO1 (Abcam) overnight at 4 °C. After incubation with an HRP-conjugated secondary antibody (Zsbio, Beijing, China), the Western blots were visualized using the enzyme-linked chemiluminescence kit (Chemicon, Temecula, CA, USA). The data analysis was performed using the Image J Software (NIH, Bethesda, MD, USA) to quantify the levels of proteins.

### Measurement of intracellular reactive oxygen species generation

For ROS staining, the cultured HCFs were washed with serum-free medium and incubated with 10 µM fluorescence probe 2,7-dichlorodihydrofluorescein diacetate, acetyl ester (DCHF-DA; Molecular Probes, Eugene, OR, USA) for 30 min at 37 °C, the unlabeled dye was washed by serum-free medium and the cells were observed under a Nikon confocal microscope. For the measurement of ROS generation, the cells were harvested and incubated with 5 µM DCHF-DA for 20 min, and then the fluorescence intensity was measured by a multimode microplate reader (SpectraMax M2; Molecular Devices, Menlo Park, CA, USA).

### Statistical analysis

The figures in this study are representative of more than 3 different experiments. Corneal injury and scarring were performed in a masked fashion. Twelve images were used for in vivo quantification in each group. Differences between the control and treated groups were analyzed using the Student’s *t* test. *P* < 0.05 was considered to be statistically significant.
